# To transform or not to transform: using generalized linear mixed models to analyse reaction time data

**DOI:** 10.3389/fpsyg.2015.01171

**Published:** 2015-08-07

**Authors:** Steson Lo, Sally Andrews

**Affiliations:** School of Psychology, University of SydneySydney, NSW, Australia

**Keywords:** RT transformations, generalized linear mixed-effect models, mental chronometry, interaction effects, additive factors

## Abstract

Linear mixed-effect models (LMMs) are being increasingly widely used in psychology to analyse multi-level research designs. This feature allows LMMs to address some of the problems identified by Speelman and McGann (2013) about the use of mean data, because they do not average across individual responses. However, recent guidelines for using LMM to analyse skewed reaction time (RT) data collected in many cognitive psychological studies recommend the application of non-linear transformations to satisfy assumptions of normality. Uncritical adoption of this recommendation has important theoretical implications which can yield misleading conclusions. For example, Balota et al. (2013) showed that analyses of raw RT produced additive effects of word frequency and stimulus quality on word identification, which conflicted with the interactive effects observed in analyses of transformed RT. Generalized linear mixed-effect models (GLMM) provide a solution to this problem by satisfying normality assumptions without the need for transformation. This allows differences between individuals to be properly assessed, using the metric most appropriate to the researcher's theoretical context. We outline the major theoretical decisions involved in specifying a GLMM, and illustrate them by reanalysing Balota et al.'s datasets. We then consider the broader benefits of using GLMM to investigate individual differences.

## Introduction

A central theme of this special issue is how the uncritical use of statistical procedures in psychological research can lead researchers to draw incorrect theoretical and practical conclusions. From a procedure as simple as averaging over a set of data points, Speelman and McGann (2013) elaborated how the resulting value is often used to draw conclusions that violate many theoretical positions describing individual, or even moment to moment, volatility in human cognitive systems.

Similarly, Trafimow (2014) expressed concern over the use of statistical techniques like related-samples *t*-tests, which appropriately assess differences between individuals (e.g., do changes in attitudes differ across people on average because of variable X), but are ubiquitously used inappropriately to address hypotheses formulated within each individual (e.g., does variable X cause a particular person's attitude to differ).

Extending this theme, we focus on another simple procedure that can lead researchers to draw misleading theoretical conclusions if applied uncritically: the routine transformation of the dependent variable to meet assumptions of normality in inferential statistics. In particular, we address issues associated with analysis of reaction time (RT) data—one of the most commonly used dependent variables in cognitive psychological research.

For over 100 years, cognitive psychologists have used RT to investigate unobservable mental processes (Donders, 1868/1969; Luce, 1986). These investigations are based on two fundamental assumptions: (i) mental processes take time to complete, and that (ii) each measured RT reflects a composite of several distinct stages of processing (e.g., visual encoding, mental processing, and response selection). This “chronometric” approach to mental processes underpins many paradigms in cognitive psychological research (Posner, 1978).

Because any single RT might contain idiosyncratic processes, such as lapses in attention, orthogonal to the mental process under investigation (however see Speelman and McGann, 2013 for an alternative perspective), researchers usually recruit multiple participants and subject them to multiple measurements of RT. This distribution of RTs obtained in simple decision tasks is invariably positively skewed. In traditional mean-based ANOVA analyses, issues regarding skew are typically ignored because the method has been repeatedly shown to be “robust to violations of normality” (e.g., Glass et al., 1972; Harwell et al., 1992; Lix et al., 1996). Consequently, many cognitive theories have been developed and validated against such mean RT data, raising many of the interpretive problems highlighted by Speelman and McGann (2013).

In response to such theoretical limitations, there have been two major developments in analysis of RT in cognitive research relevant to the themes of this issue. First, many researchers have moved “beyond mean RT” (Balota and Yap, 2011) by analysing changes in the RT distribution at a more fine-grained level in order to yield more accurate measures of group performance (Heathcote et al., 2004). Application of these procedures has allowed researchers to conduct sophisticated tests of cognitive theories that cannot be distinguished on the basis of mean RT alone (e.g., Heathcote et al., 1991; Andrews and Heathcote, 2001; Yap et al., 2009). For example, Yap et al. (2009) reported that an individual's vocabulary level modulated how word frequency and semantic priming affected the shape of their RT distribution. They found additive effects between these factors across the RT distribution for those of high vocabulary, suggesting that semantic priming was automatically triggered for both high and low frequency words among these people with highly fluent lexical representations. In contrast, those of low vocabulary showed interactive effects, particularly for slow responses, suggesting that the increased skew associated with greater priming for less familiar, low frequency target words might be due to strategic use of semantic information. Analyses of individual RT distributions have therefore proved to be useful in identifying and interpreting individual differences in speeded response tasks.

A second recent response to limitations of traditional ANOVA analyses of mean RT, which is the focus of the present paper, is the use of linear mixed-effect models (LMMs). LMMs have become increasingly prevalent within many areas of science, because they are able to account for random populations that share a nested relationship like hospitals chosen from different districts (Carey, 2002), or blocked relationships like fertilizer treatment on samples over different soil plots (Lane, 2002). Within cognitive psychology, LMMs have had the strongest recent impact in psycholinguistics, because the use of mean RT in traditional ANOVA analyses has been unable to capture the crossed relationship between counterbalanced sets of linguistic stimuli presented to different subjects (Clark, 1973; Forster and Dickinson, 1976; Baayen, 2008). LMMs provide a statistical solution to this problem (Baayen et al., 2008), and have become the recommended form of analysis in high impact journals within the field.

Importantly, LMMs have the potential to address many of the problems raised by Speelman and McGann (2013) about the use of mean RT, because the ability of these models to simulate the multi-level structure of the designs described above eliminates the need to average data across subjects, items, plots, or hospitals. This crucial property of LMMs therefore provides a powerful and refined method for investigating interactions of experimental effects with individual and item differences that cannot be investigated in traditional ANOVA approaches because they do not collapse across these variables. For example, by exploring the variance/covariance parameters, Kliegl et al. (2010) showed that individuals who responded more quickly tended to produce larger masked repetition priming effects in a lexical decision task. Across individual trials, Kinoshita et al. (2011) showed that sensitivity to the difficulty of the previous trial interacted significantly according the prime-target relationship and task environment in a parity judgment task. Thus, LMMs have the potential to accommodate the different levels of analysis required to “optimize both scientific rigor and sensitivity to individual variability” that was identified as one of the goals outlined in this Special Issue.

Although the sophistication of LMMs present a significant leap forward for individual differences research, their application is complicated for skewed dependent variables like RT because current guidelines for LMM recommend that researchers transform their RT for two reasons. The first is that skewed RT data can affect the estimate of the mean, thus distorting the outcome of statistical tests. For example, Baayen (2008) recommends transforming RT data to avoid a situation in which “just a few extreme outliers might dominate the outcome, partially or even completely obscuring the main trends characterizing the majority of datapoints” (p. 33). The second reason is that non-normally distributed residuals produced by skewed data reflect a non-constant heteroscedastic pattern that affects the precision with which the standard error of the mean is estimated (Cohen et al., 2003). Therefore, researchers are expected to use the Box–Cox procedure (Box and Cox, 1964) to identify a transformation that allows them to meet the Gaussian assumptions of normality and homoscedasticity. For RTs, the transformation that best satisfies this mathematical assumption is often the reciprocal or inverse RT (Balota et al., 2013).

### To transform or not to transform?

Unfortunately, routinely applying such transformations has important theoretical implications. For example, applying a non-linear (e.g., log, inverse) transformation to the dependent variable not only normalizes the residuals, but also distorts the ratio scale properties of measured variables, such as dollars, weight or time (Stevens, 1946). As a concrete example within the aging literature, two samples—one older and one younger—might exhibit differential benefits in RT when the preceding prime word was semantically related to the target (e.g., nurse–doctor) relative to when it was semantically unrelated (e.g., plane–doctor) (e.g., 600 and 700 ms for the younger adults, and 780 and 910 ms for the older adults). However, on the log-transformed scale, differences between these two samples are obscured because on this scale the differences disappear [e.g., log(700 ms)−log(600 ms) = 0.15415; log(910 ms)−log(780 ms) = 0.15415] (i.e., there is no interaction between age and priming).

While many readers will recognize these discrepant results as another example of “scale dependent” interactions (Loftus, 1978), the critical question that we wish to address is what the correct scale should be in “chronometric” research. According to the “mental chronometry” approach (Posner, 1978), the answer is clearly raw RT. Differences in RT over experimental conditions are assumed to directly reflect differences in the amount of time taken to perform these mental operations (Townsend, 1992). In the example above, additive effects suggest that automatic spreading activation, which is thought to underlie semantic priming, proceeds in much the same way for both younger and older adults (e.g., Hasher and Zacks, 1979), whereas over-additive effects suggest that age-related deficits in terms of response speed interacts with semantic activation in order to produce greater savings in time when both the prime and target are semantically related (Laver and Burke, 1993).

But this does not mean that raw RT is always the most appropriate dependent variable. Other theoretical positions assume a different relationship between RT and mental operations that is most appropriately measured by a transformation such as log or inverse RT. For example, differences calculated on the logarithmic metric reflect proportional change [i.e., log(700 ms)−log(600 ms) = log(700/600 ms)], which aligns with many theories of aging which attribute a causal role to general cognitive slowing (e.g., Salthouse, 1985). However, the vast majority of cognitive theories have been developed and validated on raw RT. So by routinely applying a transformation to yield the normal distribution required for LMM, the researcher may ultimately fail to test their hypotheses using the dependent variable that underpinned their theoretical predictions.

In individual differences research, scale dependent interactions touch upon even broader theoretical implications. At its most basic conceptualization in a two-factor design, a significant interaction indicates that the effect of a particular variable (the numerical difference on the dependent variable between levels of one of the factors) changes across the population of interest because it differs as a function of a second independent variable; typically another group of people or a different condition. Conversely, a lack of interaction between these factors suggests that the average effect remains uniform across individuals or conditions under assessment. Thus, statistical assessment of interactions provides insight as to whether there is a single “true value that we are trying to approximate when we measure humans on some dimension” (Speelman and McGann, 2013, p. 2), or whether multiple values exist particular to each individual.

Thus, the increasing reliance on LMM in cognitive psychology presents researchers with a conundrum created by the mismatch between the dependent variable dictated by theory and the dependent variable dictated by the requirements of the statistical analysis. As discussed above, in cognitive psychological investigations of “mental chronometry,” raw untransformed RTs are usually the metric about which the researcher has predictions. However, to satisfy the assumptions of LMM, the statistical analysis is conducted on the transformed metric. Thus, in order to interpret the results and in order to compare them with earlier published ANOVA data, the estimates of the empirical effects from the LMM are often “back-transformed” into raw RT. But unfortunately, back-transformation can be unreliable because statistically significant differences on the transformed metric are uninformative as to whether significant differences exist on the original untransformed metric and vice versa (Berry et al., 2010). Cognitive psychologists are therefore trapped between a rock and a hard place. Analyses on raw RT are inappropriate because they fail to meet the assumptions of the linear model, but analyses on transformed RT are uninformative because they fail to answer the research questions of interest.

The ideal solution to this quandary would be to allow statistical assessment on the original raw RT metric, but to also meet the mathematical constraints imposed by the statistical model. Such a solution is offered by generalized linear mixed-effect models (GLMMs) which offer one approach to achieving this ideal that is readily implemented in many statistical packages. By separating the mathematical and theoretical components of the model, GLMMs allow researchers to use the dependent variable most appropriate to their research question, while simultaneously meeting the mathematical criterion of normalized, homoscedastic residuals in linear regression. To achieve these goals, GLMMs require the researcher to consider these issues as part of the specification process.

### A case study: Effects of word frequency and stimulus quality on lexical retrieval

To demonstrate the interpretative problems associated with routinely transforming RT to meet the normality assumptions of LMM and to illustrate how GLMM can be applied to avoid the need for transformation, we present re-analyses of data recently reported by Balota et al. (2013). Specifically, they used LMM to re-analyse the data from three published studies which reported additive effects of word frequency and stimulus quality in ANOVA analyses of raw RT (Yap and Balota, 2007; Yap et al., 2008). However, for the LMM analyses on inverse RT, the data transformation that most effectively normalized the residuals for all datasets, the results yielded a completely different pattern for all three experiments: significant underadditive interactions.

In “chronometric” research, additive or interactive effects reflect fundamental assumptions about the nature of RT described at the beginning of this paper. Because each measured RT is assumed to reflect a composite of several distinct stages of processing, separate stages in mental operation can be inferred if the time required to perform a second mental operation is independent of the time required to complete the first mental operation (i.e., the effects are additive) (Sternberg, 1969). This reasoning is crucial for additive-factors logic (Sternberg, 1969), because without the ratio measurement scale properties in raw RT (Townsend, 1992), the inferential power of this technique is lost because equivalence in measurable raw RT can no longer be taken as evidence of equivalence in processing.

Thus, within the additive-factors logic (Sternberg, 1969) framework described above, the temporal relationship between word frequency and stimulus quality has important theoretical implications regarding the nature of lexical representation. Taken individually, low frequency words and visually degraded stimuli both serve to slow RT relative to when the stimuli are clearly presented or of high frequency (Stanners et al., 1975). However, the additive effects of these two variables on raw RT reported in the original papers suggest that that these factors selectively influence separate stages of mental processing, and produce significant challenges for activation models which predict interactive effects between frequency and stimulus quality (Borowsky and Besner, 1993). Specifically, activation models propose that the threshold for activation is determined by word frequency and the rate of activation by stimulus quality, so stronger effects of stimulus quality on low frequency words should therefore be observed because more time is required to reach the higher activation threshold for low frequency words when combined with a slower rate of activation in the context of degraded stimuli (Morton, 1969). This consistent evidence of additive effects of word frequency and stimulus quality in the experimental data, under conditions that yield interactions between each of these variables and semantic priming, therefore presents a strong challenge to fully interactive activation models (Borowsky and Besner, 1993; Balota et al., 2013). Given the central theoretical importance of the additive effects of word frequency and stimulus quality observed on raw RT, Balota et al.'s (2013) demonstration that the additive pattern is specific to raw RT and changes when the dependent variable is transformed directly reflects the theoretical quandary presented above.

## The generalized linear mixed-effect model (GLMM) framework

GLMMs combine and extend the properties of LMM and generalized linear model (GLM) approaches, by relaxing LMM's assumption that the dependent variable (and the residuals) follow a normal (Gaussian) distribution, and extending GLM's scope of inference to extend beyond a single random population. Rather than making the default assumptions of LMM methods, GLMM requires researchers to specify a number of components of their data and design:

1. the explanatory variables responsible for systematic variation in responses: referred to as the *fixed factors*;
2. the sampling structure of the design contributing to random variability in responses: the *random factors*;
3. the probability distribution describing the plausible processes underlying the observed data: the distribution of the *dependent variable*; and
4. the mathematical function characterizing the relationship between the fixed factors and the dependent variable: the *link function*.

The following sections introduce the key theoretical and methodological issues regarding specification of GLMMs within the context of the three experiments from Balota et al. (2013). Readers interested in more technical mathematical and computational details regarding LMM (Pinheiro and Bates, 2000; Raudenbush and Bryk, 2002; Baayen, 2008), GLM (McCullagh and Nelder, 1989), and GLMM (Jiang, 2007; Stroup, 2013) should consult the excellent resources already published on these topics.

The three experiments re-analyzed by Balota et al. (2013) each factorially manipulated word frequency and stimulus quality within a lexical decision task. For the word responses in all three experiments, each participant responded to 100 high frequency and 100 low frequency words, presented in either clear or degraded stimulus quality conditions. In Yap and Balota (2007), the stimulus quality manipulation was conducted between subjects while Yap et al. (2008, Experiments 1 and 2) used within-subjects manipulations conducted on counterbalanced item sets. The non-word items in Yap and Balota (2007) and Yap et al. (2008, Experiment 1) comprised of 200 pronounceable pseudo-words (e.g., *flirp*), while Yap et al. (2008, Experiment 2) used 200 pseudo-homophones (e.g., *brane*). Further details regarding the design are available in each experiment's respective published reports.

### The fixed factors

Users of ANOVA and ordinary least squares regression in the basic linear model framework will already be familiar with specifying fixed factors in their analyses. Both at a conceptual and practical level, this remains unchanged in GLMM. In order to differentiate them from random factors described below, fixed factors are the components of the linear predictor responsible for systematic variability in the observed responses. Typically, fixed factors consist of the independent variables (or covariates) with a small finite number of levels under experimental manipulation. The levels of these factors are the object of hypothesis testing (fixed effects), and represent the conditions for which the model provides estimates of the average response over the entire population(s) (generally denoted by the symbol $\hat{\mu }$ —the estimated mean corresponding to each condition).

Across the three experiments reported in Balota et al. (2013), the fixed factors correspond to word frequency and stimulus quality. Normalized sum contrasts specified on these fixed factors yielded four fixed effects in the statistical model: mean RT associated with the lexical decision task (intercept), differences in RT associated with the manipulations of word frequency (high vs. low), stimulus quality (clear vs. degraded), and frequency × stimulus quality interaction^1^. Of central interest is whether the observed data are consistent with interactive effects of frequency and stimulus quality predicted by interactive models, or the additive effects that follow from the independent processing stages assumed by serial models.

### The random factors

Within the mixed modeling framework, random factors correspond to components of the linear predictor in which a random subset of levels are sampled from a larger population. As opposed to fixed factors, in which systematic variability between conditions (i.e., mean differences) is explicitly estimated and compared, variability in the random factors is used to: (1) estimate the extent to which mean responses vary across units of the random factor; (2) allow inferences about whether fixed effects generalize beyond the units sampled in the random factor; (3) remove variability in responses that are associated with the random factor rather than the conditions of experimental interest (i.e., reduce Type I error rate). Typically, many levels of the random factor are sampled in the experiment under which responses are meaningfully clustered. Although clustering is one form of structural dependency typically associated with a random factor, other structural dependencies such as nesting, cross-classification, blocking and other counterbalancing procedures can also contribute to nuisance variability that is partialled out with a random factor^2^.

Subjects and items constitute the random factors common across the three experiments reported in Balota et al. (2013), because responses are clustered according to individual participants and English words—both of which represent a random sample from their respective populations. Following nomenclature within the LMM literature (e.g., Barr et al., 2013), the overall mean for each subject and item were estimated as “random intercepts” in each of the experiments, while with the degree to which each fixed effect varied across subjects and/or items were estimated as “random slopes.” This latter specification for random slopes differed according to the design of the three experiments. In the Yap and Balota (2007) experiment, stimulus quality was manipulated between-subjects and word frequency was manipulated between-items, so the random slopes controlled for subject-specific variability in the frequency effect which can be distinguished from variability associated with particular words, and item-specific variability in the stimulus quality effect which can be distinguished from variability associated with different participants. For the other two experiments in which word frequency and stimulus quality were both manipulated within-subjects, the random slopes controlled for subject-specific variability in the frequency effect, stimulus quality effect, and frequency by stimulus quality effect, as well as item-specific variability in the stimulus quality effect. This represents the “maximal” random effect structure (Barr et al., 2013) for each of the experiments.

### The dependent variable

A key feature of GLM and GLMM is the ability to appropriately model a variety of response distributions. As noted previously, GLMM does not make the default assumption that this distribution is Gaussian and therefore requires that the researcher specify an appropriate distribution. In some measurement contexts, this selection is straightforward—binary responses are described by a binomial distribution; count responses are described by a Poisson distribution. But selecting the appropriate dependent variable is less straightforward in domains like cognitive psychology, where researchers often investigate latent constructs that are indexed by continuous behavioral measures, like RT, which can be described by a host of distributions (e.g., normal, beta, gamma, uniform, etc.), and where there is often no consensus on the “correct” distribution. This ambiguity has contributed to researchers' willingness to transform RT measures to meet the mathematical assumptions of LMM. GLMM offers an alternative: the researcher can select the quantitative distribution that best captures the properties of their measured variable. As we describe below, both theoretical and empirical considerations underpin this decision.

**Figure 1:**
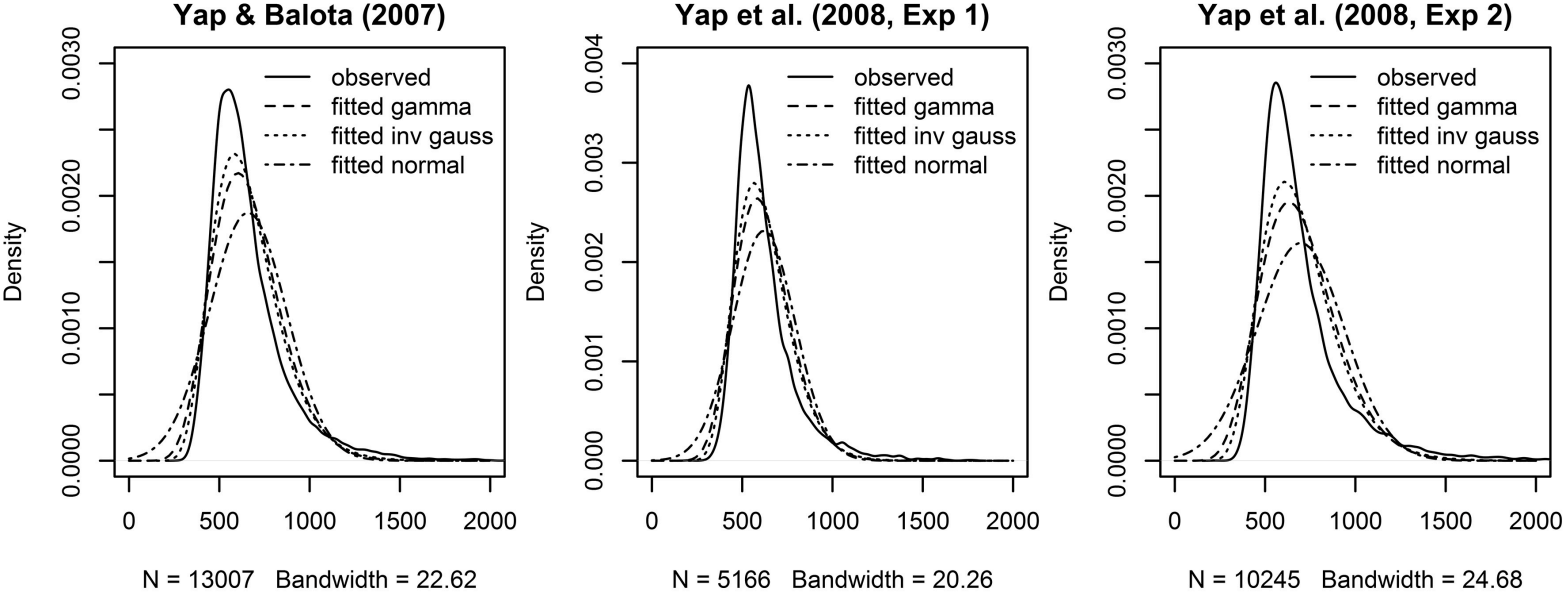
**Observed (solid lines) and fitted densities (dotted, dashed and broken lines) to the RT from the three Balota et al. (2013) experiments**. For the Yap and Balota (2007) experiment, the gamma density was fitted with a rate of 0.018 and a shape parameter of 11.967 (dashed line), the inverse Gaussian density was fitted with a λ parameter of 8201.5 and a mean of 658.8 (dotted line), the normal density was fitted with a mean of 658.8, and a standard deviation of 212.97 (broken line). For the Yap et al. (2008, Experiment 1), the gamma density was fitted with a rate of 0.026 and a shape parameter of 16.000 (dashed line), the inverse Gaussian density was fitted with a λ parameter of 10223.8 and a mean of 620.1 (dotted line), the normal density was fitted with a mean of 620.1 and a standard deviation of 172.26 (broken line). For the Yap et al. (2008, Experiment 2), the gamma density was fitted with a rate of 0.015 and a shape parameter of 10.740 (dashed line), the inverse Gaussian density was fitted with a λ parameter of 7704.3 and a mean of 697.0 (dotted line), the normal density was fitted with a mean of 697.0 and a standard deviation of 242.67 (broken line).

Across the three experiments reported in Balota et al. (2013), the dependent variable was the RT to correctly classify each stimulus as an English word. As illustrated in Figure 1, the distributions of observed RT (represented by solid lines) for all three experiments were unimodal with a distinct positive skew. In addition to this characteristic shape, the data for all experiments also revealed a linear relationship between the standard deviation of RTs and mean RT demonstrated in many previous studies of RT in binary choice tasks (e.g., Luce, 1986; Faust et al., 1999; Wagenmakers and Brown, 2007). This linear relationship is also evident in plots of the residuals which show hetereoscedasticity in LMM analyses, evidenced by increasing spread in residuals for longer predicted RT (Kliegl et al., 2010; top row of plots in **Figure 3**).

Rather than transforming the dependent variable to eliminate this deviation from normality, GLMM allows the researcher to select a theoretical distribution that matches the properties of measured RT. Two of the two-parameter distributions currently implemented for GLMMs in the stats package as part of the default installation of the R program for statistical computing (R Core Team, 2013), the Gamma and Inverse Gaussian distributions reproduce these surface characteristics of raw RT—a unimodal skewed distribution with continuous responses greater than or equal to 0. As shown in Figure 1, they both provide a closer approximation to the observed distribution of RTs in the three experiments than the normal distribution. The distributions also provide an explicit mathematical relationship between the mean and variance. For the Gamma distribution, the variance of the distribution increases proportionally with the mean, while the variance increases proportionally with the cube of the mean for the Inverse Gaussian distribution. Despite the differences in their mathematical expression, both distributions are able to approximate a variety of distributional shapes that allow them to “statistically mimic” RT responses and yield fits that are practically indistinguishable from each other (Van Zandt and Ratcliff, 1995).

As well as approximating the surface characteristics of the distribution of the dependent variable, the probability distribution should also provide a plausible description of the processes underlying the response. At a conceptual level, both the Gamma and Inverse Gaussian distributions can be linked to *waiting time*—how long it takes until an event of interest (e.g., a button press) to occur. Mathematically, the Gamma distribution is the sum of multiple exponential distributions, which can be considered to model the probability that no event occurs until a certain period of time. The Gamma distribution can therefore be considered to model several serial stages of processing, each of which finishes with a time that is exponentially distributed (Van Zandt and Ratcliff, 1995). Similarly, the Inverse Gaussian distribution has been identified with the time for evidence accumulation to reach a single threshold boundary within a diffusion process (Schwarz, 2001). There are other distributions as described in the General Discussion (e.g., ex-Gaussian, ex-Wald, shifted Wald) with parameters that have also been associated with psychological processes underlying RT (Matzke and Wagenmakers, 2009). Given that there is no consensus as to the “correct” distribution for mapping from psychological processes to RTs, the purpose of this introduction is not to advocate for a particular distribution, but to illustrate that the Gamma and Inverse Gaussian are examples of distributions that provide a plausible description of processes reflected in RT.

### The link function

In GLM and GLMM, fixed factors are assumed to be linear predictors of a function of the observed response rather than the observed response itself. Thus, the model assesses the linear predictors ($\hat{\mu }$) on an *unbounded transformed scale* (e.g., the scale upon which a latent variable like “lexical retrieval” is measured could contain any numerical value), that is different from the *bounded original scale* of the dependent variable (DV) (e.g., observed RT contains strictly positive values like those produced by the Gamma distribution; the observable probability of an inaccurate response is bound between the values of 0 and 1 like those from a binomial distribution). The transformed and original scales are connected by a monotonic differentiable link function that allows back-transformation to the original metric by providing a one-to-one mapping between the range of fitted values produced by the linear predictor on the transformed metric and the range of observed values on the original metric [i.e., DV = *f* ($\hat{\mu }$)]. Therefore, the nature of the relationship between the two scales can be considered to be defined by the mathematical function connecting the observed response to the latent construct upon which the fixed factors are assessed. In the special case where “no function” is required and the observed response is assumed to directly tap the latent construct (e.g., RT is a direct measure of the time required for lexical retrieval), the function binding the expected values produced by the predictors to the dependent variable is the *identity link* (i.e., DV = $\hat{\mu }$). Ordinary linear regression and LMM assumes an identity link between the DV and the latent construct. When researchers using these methods believe that the measured DV is not directly related to the latent construct, they can mathematically transform the DV into the latent construct, and then apply this transformed variable in the analysis as the DV in order to achieve a similar effect^3^. That is, the link function in GLM(M) explicitly defines the nature of the expected relationship between the predictors and the observed response.

In the context of the experiments reported in Balota et al. (2013), there are two reasons as to why the identity link is appropriate. Firstly, from a theoretical perspective, the tradition of mental chronometry assumes that manipulations directly affect RT rather than some function of RT. More explicitly within additive factors logic, RT is assumed to be linearly affected by the experimental factors so that factors that affect a single processing stage interact, while those that affect separate processing stages do not. By changing the form of this mapping with a non-linear link function or a non-linear transformation of the dependent variable as applied in LMM, such an interpretation cannot be applied and cannot inform the models from which they were derived. Secondly, from a mathematical perspective, a non-linear link function is usually applied to constrain the predicted values within the bounds of the dependent variable. Since the bulk of observed RTs in Balota et al. (2013) are situated well away from the negative boundary (in part because RTs faster than 200 ms were removed), and predictions are not extrapolated beyond the observed conditions, there is little danger of the model producing impossible negative values for RT which are difficult to interpret.

## Using GLMM to avoid the need for transformation of skewed RT data

To illustrate the application of GLMM to address the problems with transformation outlined earlier, we re-analyzed the three experiments that Balota et al. (2013) recently demonstrated to yield contradictory outcomes in analyses conducted on raw and transformed data. They report that LMM analyses of the inverse RT transformed data that best satisfied criteria for normality yielded underadditive interactions rather than the additive effects of frequency and stimulus quality found with raw RT.

We report the results of six analyses of each of the three experiments. Two of the analyses parallel Balota et al.'s (2013), by using LMMs on raw RT (DV = RT) and inverse RT (DV = −1000/RT). By default, these analyses assume a Gaussian distribution and identity link function. The remaining four analyses are GLMMs on raw RT which assume either a Gamma or Inverse Gaussian distribution of the DV, and a linear (identity link function; RT = $\hat{\mu }$) or inverse relationship (inverse link function; RT = −1000/$\hat{\mu }$) between the predictors and RT. We chose −1000/$\hat{\mu }$ as the specific form of the inverse link function to parallel the inverse transformation applied to RTs in Balota et al.'s (2013) LMM analyses (i.e., −1000/RT). These GLMM analyses are therefore analogous to the LMM analyses conducted on inverse RT.

The primary interest is in the results from the properly specified GLMM based on the decisions described in the previous section, but we also aim to clarify how differences in the specification of the dependent variable and link function relate to the conflicting findings between raw and inverse transformed RT reported by Balota et al. (2013).

The analyses were conducted on RT data for correct word responses for Yap and Balota (2007) and Yap et al. (2008 Experiments 1 and 2) using version 1.0-5 of the lme4 package (Bates et al., 2013) in the R program for statistical computing (R Core Team, 2013) following the same trimming procedures described in Balota et al. (2013). Since there is continuing debate as to how *p*-values should be generated for LMMs because of computational issues regarding degrees of freedom, we follow the current practice of considering effects greater than two standard errors (i.e., |*t*|> 2) to be significant at the 0.05 level for datasets involving a large number of observations (Kliegl et al., 2010; Masson and Kliegl, 2013). The R syntax used to generate these models along with the full model output and predicted mean RT for each condition can be found in the Supplementary Materials.

**Figure 2:**
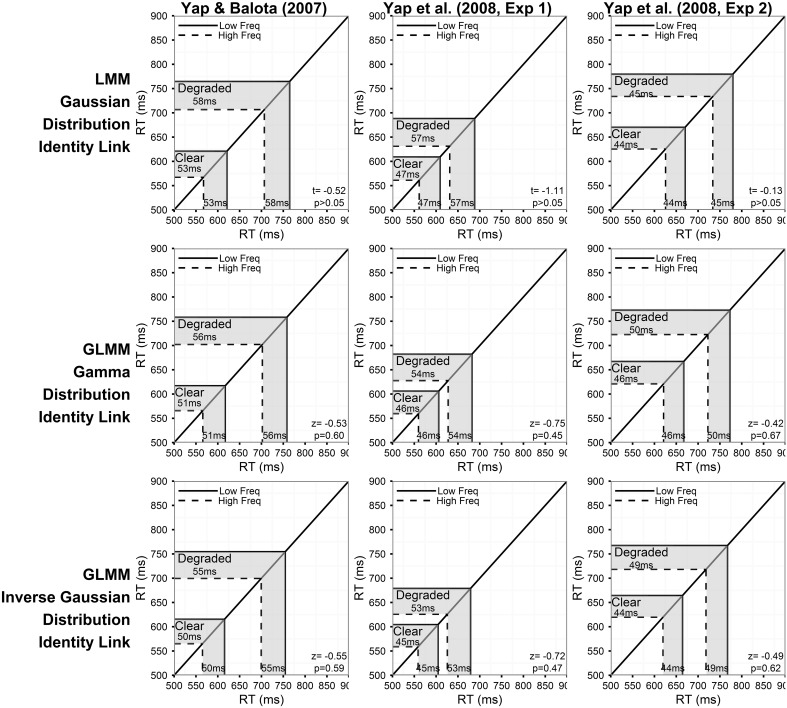
**Prediction plots illustrating the estimated frequency effect and statistical results (***t***- or ***z***- value and corresponding ***p***-value) of the word frequency by stimulus quality interaction (shaded region on x-axis) based on models assuming a linear relationship between the predictors and RT (identity link function)**. Note that the back-transformed estimates (shaded region on y-axis) are identical because of the identity link function. Each column of plots represents the results from a different experiment (from left to right: Yap and Balota, 2007; Yap et al., 2008: Experiment 1; and Yap et al., 2008: Experiment 2), and each row of plots represents a different assumption for the distribution of RTs (from top to bottom: Gaussian, Gamma, and Inverse Gaussian). Note that precise *p*-values are produced in GLMM for the Wald Z-statistic in R, while approximate *p*-values can only be inferred based on the magnitude of the *t*-value produced in LMM.

**Figure 3:**
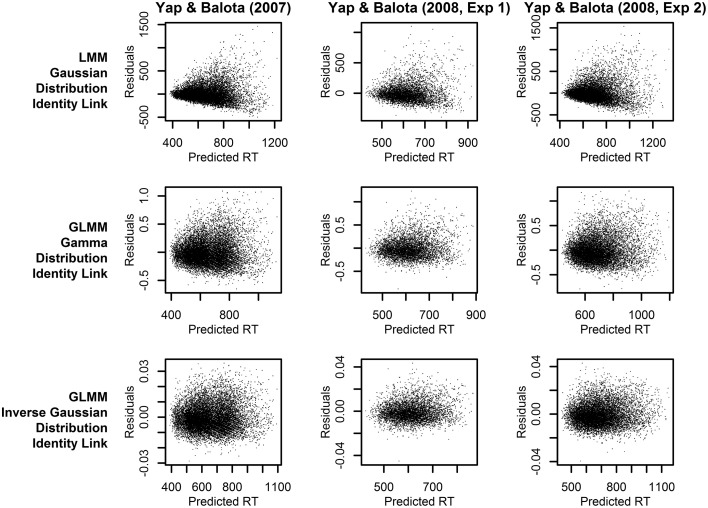
**Plots of the residuals over predicted RT from models assuming a linear relationship between the predictors and RT (identity link function)**. Each column of plots represents the results from a different experiment (from left to right: Yap and Balota, 2007; Yap et al., 2008: Experiment 1; and Yap et al., 2008: Experiment 2), and each row of plots represents a different assumption for the distribution of RTs (from top to bottom: Gaussian, Gamma, and Inverse Gaussian).

**Figure 4:**
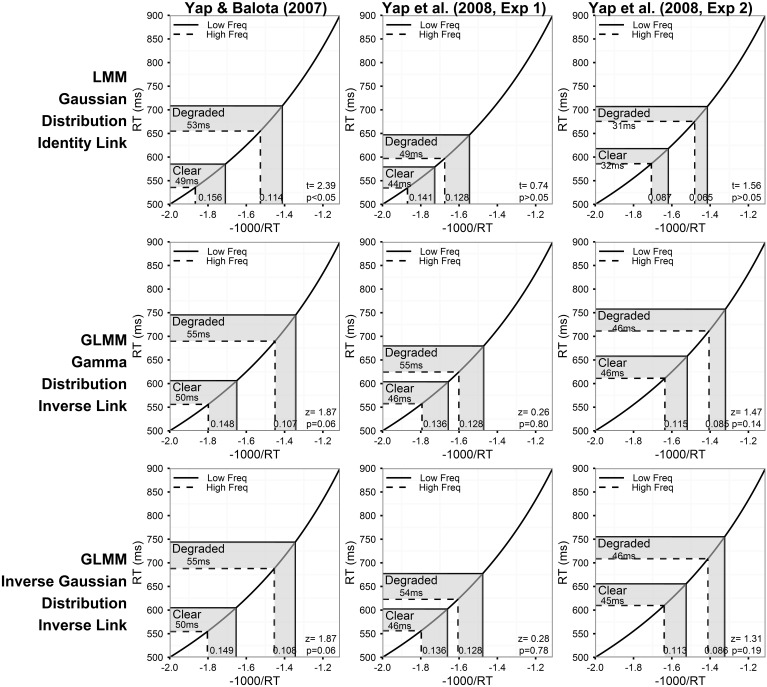
**Prediction plots illustrating the estimated frequency effect and statistical results (***t***- or ***z***-value and corresponding ***p***-value) of the word frequency by stimulus quality interaction (shaded region on x-axis) based on models assuming an inverse relationship between the predictors and RT (inverse link function)**. The plots also present the back-transformed estimates (shaded region on y-axis) on the original RT metric. Each column of plots represents the results from a different experiment (from left to right: Yap and Balota, 2007; Yap et al., 2008: Experiment 1; and Yap et al., 2008: Experiment 2), and each row of plots represents a different assumption for the distribution of RTs (from top to bottom: Gaussian, Gamma, and Inverse Gaussian). Note that precise *p*-values are produced in GLMM for the Wald Z-statistic in R, while approximate *p*-values can only be inferred based on the magnitude of the *t*-value produced in LMM.

**Figure 5:**
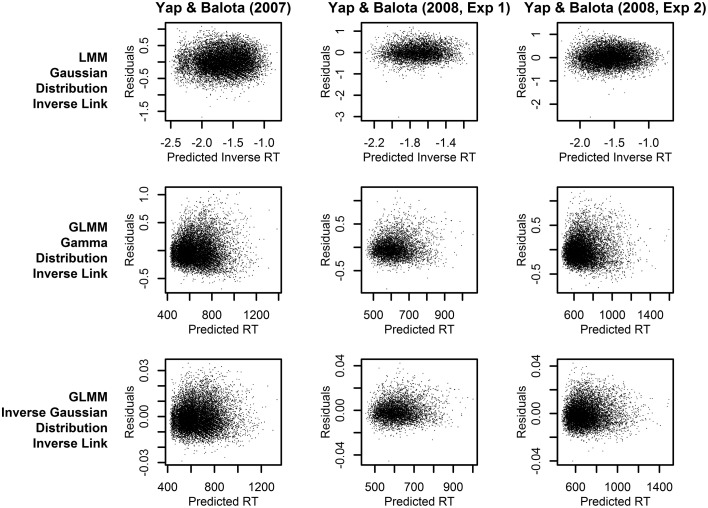
**Plots of the residuals over predicted RT (or inverse RT) from models assuming an inverse relationship between the predictors and RT (inverse link function)**. Each column of plots represents the results from a different experiment (from left to right: Yap and Balota, 2007; Yap et al., 2008: Experiment 1; and Yap et al., 2008: Experiment 2), and each row of plots represents a different assumption for the distribution of RTs (from top to bottom: Gaussian, Gamma, and Inverse Gaussian).

Figure 2 summarizes the predictions of the models assuming a linear relationship between the predictors and RT for the three experiments. The corresponding results for models assuming an inverse relationship between the predictors and RT are presented in Figure 4. Each column of Figures 2–5 corresponds to a different experiment, while the rows of the figures present estimates from the LMM models (top row), and GLMM models assuming Gamma (middle row), and Inverse Gaussian (bottom row) distributions, respectively, of the DV.

For each model summarized in Figures 2, 4, the shaded region of the prediction plot depicts the estimated effect of word frequency (difference between high and low frequency conditions) based on the fitted values for each of the four frequency by stimulus quality conditions as plotted on the model transformed scale (x-axis), while the y-axis plots the same difference after the mean estimates have been back-transformed via the link function on the original RT scale. The estimates are identical on the model and back-transformed RT scales in Figure 2 because the identity link assumes that the scale of the latent construct assessed by the model (x-axis) is synonymous with RT. The form of the link function itself is depicted by the solid black line connecting the diagonals of the plot.

Although an identity link function (DV = $\hat{\mu }$) was also specified for the LMM analysis on inverse transformed RTs (DV = −1000/RT), we depict a non-linear function in Figure 4 to illustrate the back-transformation from inverse to raw RT (RT = −1000/$\hat{\mu }$) that researchers typically apply to interpret their data. The *p*-value corresponding to the critical interaction effect, which is presented in the bottom-right corner of each plot only assesses whether there is a significant difference in the linear effect of frequency on the model transformed scale (x-axis), and does not assess whether significant (linear) differences exist on the original RT scale (y-axis) unless the identity link was specified (Berry et al., 2010).

### Selecting the right model

Each of the individual analyses in Figures 2, 4 produced subtle differences in the magnitude, direction or statistical significance of the word frequency by stimulus quality interaction. A decision must therefore be made about the best-fitting correctly specified model. There are a number of ways to address this question.

Throughout the previous sections, we have argued that, from a theoretical perspective, the dependent variable of theoretical interest in mental chronometric research like this is raw RT, and that additive factors logic assumes a linear relationship between the experimentally manipulated variables and RT itself. From this perspective, only the analyses using raw RT as the dependent variable and specifying an identity link function provide meaningfully interpretable results for this experiment (Figure 2).

To further discriminate between the analyses, we can identify the statistical model that provides predictions which best fits the observed RTs. Figure 3 allows a visual inspection of model fit, by plotting the residuals against predicted RT. The LMM analyses (top row of plots), which assume a Gaussian distribution of raw RT, clearly exhibit a heteroscedastic (fan-shaped) pattern that is not evident in the GLMM analyses assuming a Gamma or Inverse Gaussian distribution (middle and bottom row of plots). Therefore, these plots suggest that the Gamma or Inverse Gaussian distributions provide a better fit to the data because they explicitly account for the hetereoscedastic pattern of increasing variability with slower responses and therefore yield more normally distributed residuals.

A similar conclusion derives from AIC and BIC summary fit indices presented in Table 1, and the estimated Gaussian, Gamma, and Inverse Gaussian distribution fits to the observed RT density in Figure 1. Across the three experiments, the Inverse Gaussian distribution (followed by the Gamma and Gaussian distributions) produce parameters that best approximate the shape of the observed RT distribution, and yield fit values that are consistently lower than the Gamma or Gaussian distributions. Thus, on both these graphical and empirical indices, the Inverse Gaussian distribution provides the best fitting model.

**Table 1:** **AIC and BIC indices of model fit comparing LMMs and GLMMs of different distribution and link assumptions for each of the three experiments**.

**Link function**	**Distribution (DV)**	**Yap and Balota (2007)**		**Yap et al. (2008, Experiment 1)**		**Yap et al. (2008, Experiment 2)**	
		**AIC**	**BIC**	**AIC**	**BIC**	**AIC**	**BIC**
LMM (Identity link)	Gaussian (inverse RT)	6337	6404	3284	3356	6832	6912
	Gaussian (raw RT)	170,573	170,640	66,775	66,847	138,196	138,276
GLMM (Identity link)	Gamma (raw RT)	164,722	164,790	64,954	65,026	133,528	133,608
	Inverse Gaussian (raw RT)	163,161	163,229	64,461	64,533	132,318	132,398
GLMM (Inverse link)	Gamma (raw RT)	164,545	164,613	64,870	64,942	133,304	133,384
	Inverse Gaussian (raw RT)	163,012	163,079	64,395	64,467	132,128	132,207

*Note that the dependent variable (DV) specified in the first row (LMM) were on inverse transformed RT, so these fit indices are not directly comparable with the other five rows of models which used raw RT as the DV*.

Having identified the most appropriate statistical model, we can consider its results. Consistent with the ANOVA analyses reported in the original published papers, none of the three experiments yielded a significant interaction between word frequency and stimulus quality in the Inverse Gaussian GLMM with identity link function (bottom row of plots in Figure 2). This model predicted effects of frequency that were 5, 8, and 5 ms greater for the degraded than clear condition in the Yap and Balota (2007), Yap et al. (2008, Experiment 1), and Yap et al. (2008, Experiment 2) data, respectively. The magnitude and direction of these effects are essentially identical to the 6, 7, and 5 ms overadditive effect reported in original ANOVA analyses. Although these estimated effects are similar to those predicted in the poorer fitting Gamma and Gaussian GLMM with identity link (top and middle row of plots in Figure 2), the test statistic (*t*- or *z*-value) is larger and corresponding *p*-value lower for the better fitting models, suggesting that the standard errors have been more precisely estimated. Better fitting models provide more powerful adjustment to extreme values, particularly in the slowest condition of degraded low frequency words, where calculation of the average would be most affected, thus allowing greater power as well as reliability with which to assess individual differences between subjects and items (see Appendix in Supplementary Material for mean RT predicted for each condition by the six models).

Different conclusions about the relationship between word frequency and stimulus quality are suggested by the results of models using transformed RTs or link functions that assume a non-linear relationship between the predictors and RT. From the perspective of model fit alone, the analysis on inverse transformed RT produces residuals that offer the least amount of hetereoscedasticity (Figure 5), suggesting that the fit is at least as good, if not better, than the Inverse Gaussian GLMM with identity link described above^4^. This is the expected outcome of applying the Box–Cox procedure to estimate a power transformation that stabilizes variance in order to create normally distributed data. However, although these models meet the mathematical assumptions of normality required by LMM, as Balota et al. (2013) report, relying on the transformed DV in LMM put the researcher in the unhappy situation of developing an *ad-hoc* explanation of why the estimated effect of frequency is now underadditive (Figure 4), as opposed to the additive or slightly overadditive effects observed on raw RT.

These contradictions arise because interval differences in the dependent variable are distorted when non-linear transformations are applied. For each of the prediction plots based on an inverse transformation or inverse link function in Figure 4, almost all of the back-transformed estimates suggest no difference, or a slightly larger numerical effect of frequency for degraded words (a small overadditive effect) on the RT scale (y-axis). However, on the model estimate scale (x-axis), these differences are distorted by the non-linear inverse link function into a numerically larger effect of frequency for clear words (underadditive effect). For the Yap and Balota (2007) experiment, the distortion caused by the non-linear transformation was severe enough to push the underadditive effect to statistical significance in the LMM analysis (top left panel of Figure 4). The underadditive interactions in this dataset were also marginally significant in the GLMM analyses using the inverse link function.

To meaningfully interpret this underadditive effect, and effects assessed on the inverse RT scale more generally, the researcher must assume that the predictors are inversely related to RT. This view is consistent with recent attempts to map effects assessed on the reciprocal scale to differences in processing rate or processing speed (Kliegl et al., 2010). For example, processing rate or speed of evidence accumulation is assumed to be slower for visually degraded as opposed to clearly presented words in activation models (e.g., McClelland and Rumelhart, 1981), thus yielding the slower RT typically observed for these conditions. However, a core assumption within all of these models is that rate of evidence accumulation is linear over time (e.g., Borowsky and Besner, 1993; Ratcliff and Rouder's, 2000, diffusion model; Brown and Heathcote's, 2008, linear ballistic accumulator)—in direct contrast to the non-linear relationship implied by the inverse scale. So while there may be physiological reasons to expect non-linearity at the level of neural spike rates (e.g., Carpenter and Williams, 1995), the implications associated with the reciprocal nature of this transformation on RT appears to be limited because psychological models assuming linearity are able to closely predict responses in observed data (Ratcliff, 1978; Brown and Heathcote, 2008).

Thus, the GLMM procedure allows researchers to select the DV most appropriate to their research question rather than use a transformed DV simply to meet mathematical assumptions. If raw RT is the most appropriate metric, as we have argued to be the case for most mental chronometric research, an Inverse Gaussian or Gamma distribution can be assumed to achieve more normal homoscedastic residuals, while retaining raw RT as the DV. As Figure 2 shows, this produces more power than LMMs conducted on raw RT. Alternatively, if the researcher's predictions are for a transformed scale, such as inverse RT, specifying a non-linear link function of the same form as the inverse transformation applied to RTs (inverse link function; −1000/$\hat{\mu }$) produces an identical distortion of frequency effects toward underadditivity (see middle and bottom row of prediction plots in Figure 4). Moreover, there appears to be no loss in model fit relative to the matching models using an identity link according to both a visual inspection of the residuals (Figures 3, 5) and empirical fit statistics (Table 1).

In summary, GLMMs allow assumptions regarding the relationship between the predictors and the dependent variable to be tested independently of assumptions regarding the distribution of dependent variable. In LMM, the two are confounded because the relationship between the predictors and the dependent variable is dictated by the transformation selected to normalize the distribution of the dependent variable. By contrast, GLMM allows the form of the link function to be determined by the theoretical issues under consideration.

## General discussion

The broad goal of this paper is to echo Speelman and McGann's (2013) cautions about the routine use of statistical procedures without reflecting on the theoretical assumptions underlying their use. Within cognitive psychology, researchers are keenly aware of the dangers associated with relying on the mean, and many have begun to turn to the multilevel properties of LMMs as a way of simultaneously controlling for (or explicitly investigating) individual sensitivity between each item or participant as an explanation of overall differences between conditions (Clark, 1973; Locker et al., 2007). These methods offer one approach to reconciling the logic of group-based and individually focused research, one of the topics suggested for this Special Issue.

However, this change in statistical practice raises a new set of theoretical assumptions that have to be critically evaluated. Many cognitive researchers have adopted LMM because it is the statistical technique in current vogue, and a vast majority follow the recommendation to normalize RTs without proper consideration of the implications of such transformation for the theoretical rationale underpinning their research question. While for some researchers, the issues and recommendations proposed in this paper seem as obvious to those provided by Speelman and McGann (2013) with respect to the mean, we hope for many others that this discussion will serve as a timely reminder to reflect on the theoretical implications wedded to a seemingly innocuous statistical procedure.

Specifically, we have argued that raw RT is the most appropriate metric from the assumptions derived as part of the “mental chronometry” approach. However, transforming the dependent variable might be more appropriate from other theoretical perspectives. For example in the aging literature, theories of general cognitive slowing (e.g., Salthouse, 1985) propose that larger differences in RT for older as opposed to younger adults arise simply because the older adult's slower responses allow more time for the experimental effect to manifest (e.g., Kliegl et al., 2010). Such models therefore predict that the magnitude of effect expressed by younger and older adults should be defined by a constant ratio across RT (Myerson et al., 1992). Returning to the semantic priming example presented in the introduction, we showed that proportional differences can be mathematically expressed through logarithms. Thus, at a conceptual level, log RT is more appropriate than raw RT if one's research question is concerned with whether an experimental effect deviates from the theoretically defined proportional increase expected for slower responses. In our semantic priming example, parallel analyses of log and raw RT would therefore provide useful complementary insight regarding the nature of the relationship between response speed, age, and lexical activation.

There are, however, two major obstacles which impede the widespread application of logarithmic transformations within psychological data. The first is the finding in large-scale meta-analyses that proportional effects predicted by models such as general cognitive slowing are not fully captured by a logarithmic transformation alone, (e.g., Chapman et al., 1994; Faust et al., 1999). This is echoed in applications of the Box–Cox procedure in LMM analyses which typically identify the reciprocal rather than natural logarithm as the transformation best suited for psycholinguistic data (Balota et al., 2013). The result is that comparisons using LMM are being conducted on the inverse scale rather than on log or raw RT for which the researcher has predictions. By separating the mathematical issues related to the distribution of RT in GLMM, the researcher is able to specify the form of the link function (e.g., log, identity) that directly addresses their theoretical questions of interest.

The other major goal of the present paper is to introduce how GLMMs might be specified using a popular statistical program and concrete psycholinguistic example (see Appendix in Supplementary Material). Using a GLMM that fulfilled the mathematical requirements of homoscedastic residuals by assuming an Inverse Gaussian distribution but maintained the theoretically relevant dependent variable through the identity link function, the results yielded additive effects of word frequency and stimulus quality across the three experiments from Balota et al. (2013). This finding is important for two reasons. Computationally, the more powerful GLMM analyses yield statistical outcomes that confirm the robust additivity reported between these factors in previous literature, and yield numerical results that are consistent with a small overadditive effect estimated in the ANOVA analyses conducted by Yap and Balota (2007) and Yap et al. (2008). Theoretically, additive effects are consistent with separate stages of processing within the additive-factors framework (Sternberg, 1969) and support interpretations that assume an initial perceptual normalization process that is sensitive to stimulus quality which precedes the memory retrieval process responsible for effects of frequency (Borowsky and Besner, 1993; Yap and Balota, 2007).

**Figure 6:**
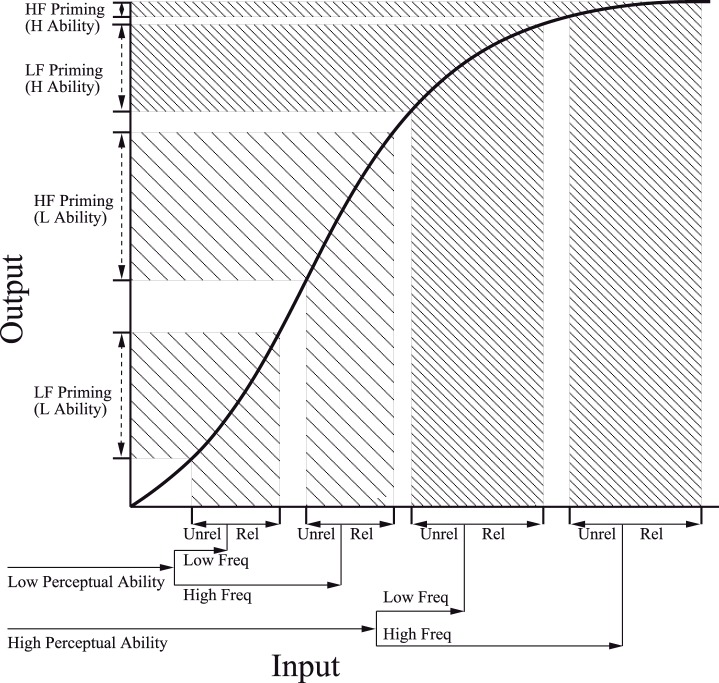
**The sigmoid activation function from the Plaut and Booth (2000) model**. In this diagram the effects of perceptual ability, word frequency, and semantic priming are all assumed to lie in the linear and upper portion of the curve. From “Individual and developmental differences in semantic priming: Empirical and computational support for a single-mechanism account of lexical processing” by Plaut and Booth (2000). Copyright 2000 by American Psychological Association. Reproduced with permission.

Alternatively, additive effects of word frequency and stimulus quality can be accommodated in dynamic connectionist models (e.g., Plaut and Booth, 2000). A core assumption underlying these models is that the amount of activation required for the network to settle and output a RT response depends on the strength of its input along a non-linear sigmoidal function (see Figure 6). Variables which produce stronger input (e.g., higher frequency words, more semantically related concepts, older individuals with greater reading or perceptual ability) elicit stronger activation within the network, and thus output faster RT. However, proportionally smaller differences on RT are expected if all of the input falls within the upper and lower extremities of the sigmoid for which RT is most compressed (right part of Figure 6), relative to the more linear middle portion of the activation curve (left part of Figure 6). As described above, this proportional difference can be mathematically defined through a non-linear transformation. For example, a reciprocal relationship between input and RT (i.e., RT = −1000/$\hat{\mu }$ as in Figure 4) might characterize a situation in which the input strength associated with word frequency and stimulus quality are both assumed to fall at specific points within the lower rising part of the sigmoid. But in order to yield the observed additive effect on RT, a smaller effect of frequency must have arisen among the clearly presented items, which are assumed to produce stronger input. Given the positive relationship between input and activation, this finding is exactly opposite to that predicted by activation models as described in the Introduction.

Conversely, a completely opposite pattern is derived if the effects of word frequency and stimulus quality are both assumed to fall on the upper part of the sigmoidal function (as depicted in Figure 6). For example, specifying a logarithmic link function [RT = 500 × log($\hat{\mu }$)], paralleling the upper section of the sigmoid function within GLMM analyses assuming an Inverse Gaussian distribution of RT, revealed a trend toward significant overadditive interaction in all three experiments (*z* = −1.75, *p* = 0.08, for Yap and Balota, 2007; *z* = −1.26, *p* = 0.21, for Yap et al., 2008 Experiment 1; *z* = −1.45, *p* = 0.15; for Yap et al., 2008 Experiment 2). Individuals can therefore yield underadditive, additive or overadditive effects depending on their hypothesized position on the sigmoidal function.

As a concrete demonstration of this possibility, Plaut and Booth (2000) hypothesized that children of both high and low perceptual ability lie within the more linear portion of the sigmoid, because these less proficient readers are understood to possess generally weaker input than highly proficient adult readers. The result is that the magnitude of semantic priming is approximately equal for both high and low frequency words among those of high or low perceptual ability. In contrast, adult readers are hypothesized to possess greater input strength, positioning them within the upper part of the sigmoid. Because of the non-linearity associated with this upper portion of the curve (see Figure 6), adult readers of greater perceptual ability produce attenuated effects of semantic priming for high frequency words, relative to the more additive effects observed among adults of low perceptual ability. By manipulating overall input strength associated with children and adults though the stimulus-onset asynchrony (SOA) of the prime, Plaut and Booth were able to induce interactive effects between semantic priming, word frequency, and perceptual ability in children by lengthening prime SOA, and more additive effects between these variables in adults by shortening SOA. Thus, Plaut and Booth's approach provides important theoretical insight into how a single mechanism (prime SOA) can yield a range of different behavioral outcomes for different individuals. However, without concrete specification of how the sigmoid maps onto the RT scale for the lexical decision task, connectionist models become unfalsifiable if the theory is able to simultaneously predict every form of relationship between the factors, and the empirical data can be transformed by different parts of the sigmoidal function to produce any pattern of effect.

In general, we recommend against a “trial-and-error” approach to specification of the link function without firm theoretical guidance. However, such an approach might be considered if the statistical analysis has the truly exploratory goal of providing a description of how the dependent variable is affected by the predictors^5^. Critically, the focus of such exploratory analyses should not be on the statistical outcome of the fixed factors, because such tests assess how much the predictors affect the transformed metric rather than the dependent variable (Berry et al., 2010). Instead, the emphasis should center on how closely the description defined by the link function fits the observed data. Interestingly, the fit values determined by the AIC/BIC criteria favor the inverse link function over the identity link for all three experiments. Since we know of no current theory that explains why word frequency and stimulus quality are defined by an inverse relationship with RT, the fact that such a relationship is observed in the data remains of interest for future theoretical development.

Besides the mathematical form of the link function, we have also emphasized the importance of specifying an appropriate probability distribution for the dependent variable. Principally, this was achieved though theoretical consideration of the processes described by the probability distribution (e.g., RTs are more likely to reflect waiting time captured by a Gamma or Inverse Gaussian distribution than the number of times an event occurs in a Poisson distribution—even though the likelihood of observing extreme responses from both these processes are positively skewed). When multiple distributions provide equally plausible description of the processes underlying the dependent variable, as is the case with RT, the statistical analysis should be conducted using each of the distributions, with final selection based on the distribution that provides the closest fit to the observed data as determined by AIC/BIC fit statistics. Although the Inverse Gaussian distribution provided a superior fit for the experiments reported in Balota et al. (2013), the Gamma or other distributions not yet considered may provide a better match for other RT experiments.

Specifically, Rouder (2005) proposed that distributions for RT should also account for differences in minimum RT across experiments or individuals. Two-parameter distributions are ill-fitting because a third “shift” parameter is thought to be necessary in order to capture the fact that there is little or no mass below this minima in observed RTs. However, three-parameter Gamma or Inverse Gaussian distributions, which are similar to the shifted lognormal or shifted Weibull used by Ratcliff and Murdock (1976) and Rouder et al. (2008), are beyond the scope of GLMMs. This has led Rouder and colleagues to develop hierarchical models that use Bayesian statistics to make the necessary computations tractable (e.g., Rouder and Lu, 2005). Although such innovations will produce significant improvements over model fit as Bayesian techniques become better supported in popular statistical programs, the same careful consideration of the relationship between RT and the linear predictors (e.g., Rouder et al., 2008), and appreciation of models that capture rather than transform the attributes of RT are issues which remain pertinent for hierarchical Bayesian models.

While the results from the Balota et al. (2013) data suggest that better fitting distributions produce more precise standard errors and statistical greater power, the statistical outcomes from these datasets also seem to be relatively robust against moderate misspecification of the distribution in the GLMM framework. Given there is now evidence that experimental factors can produce isolated or even opposing effects on different parts of the RT distribution (e.g., Heathcote et al., 1991), GLMM analyses could be supplemented by consideration of how distributional shape is affected through variation in its parameters. An important step in this direction are the distributional analyses reported in Yap et al. (2009) that demonstrated differential effects of the experimental factors on the skewed tail of the RT distribution. By fitting ex-Gaussian distributions to the observed RTs, Yap et al. (2009) detected a significant four-way interaction between an individual's vocabulary ability, word frequency, non-word type and semantic priming on the τ parameter, reflecting stronger growth in the expression of semantic priming across the RT distribution for low compared to high frequency words particularly among those of lower vocabulary scores within a pseudo-homophone non-word environment. Importantly, transforming the data and analysing log or inverse RT would have obscured these findings of variation across individuals because the slowest condition - reflecting precisely those responses from low frequency words by those of poor vocabulary in a difficult pseudo-homophone non-word environment at the very tail of the distribution—would be more affected by the non-linear transformation than any of the other conditions (Balota et al., 2013). To extend these findings, future analyses could investigate these differences within the μ or λ parameters of the Inverse Gaussian distribution used in the present analyses, or to consider effects in three parameter distributions such as the ex-Gaussian or shifted Weibull (Rouder et al., 2008).

In summary, researchers are keenly aware of the potential biases associated with using skewed RT data for mean-based analyses. This has prompted recommendations to “transform away” these “erroneous…deviations from nature's ideals” (Speelman and McGann, 2013, p. 2), which exert even greater “undue influence” in skewed data than if responses had been normally distributed. By accommodating the shape of the skewed RT distribution, GLMMs remove the need to transform the dependent variable and allow the researcher to construct statistical models that answer their questions of interest, rather than being forced to change their question of interest to meet the constraints of the statistical model. Apart from alerting researchers to the problems associated with transforming their data and potentially obscuring systematic differences between individuals, the primary focus of this paper is to introduce an alternative solution and to describe the set of decisions required to correctly specify a GLMM. We have argued that the mental chronometry assumptions underlying much of the cognitive psychological research using RT data mean that the “correct metric” to analyse is often raw RT, but have illustrated scenarios for which transformed data might be more appropriate depending on the research question at hand. Should researchers have a clear theoretical basis for expecting a non-linear relationship between the predictors and the dependent variable, we have shown how specification of the form of the link function is able to achieve the same result in GLMMs without directly transforming the raw data. As the present analyses demonstrate, without such theoretical motivation, analyses based on non-linear transformations can lead researchers to spuriously conclude that an average effect is uniform across individuals or conditions (or vice versa) by altering the scale of the differences in an interaction to produce misleading or potentially contradictory results.

### Conflict of interest statement

The authors declare that the research was conducted in the absence of any commercial or financial relationships that could be construed as a potential conflict of interest.

## Footnotes

^1^Balota et al. (2013) also included the lexicality and stimulus quality of the previous trial as fixed factors in their analyses in order to investigate the modulating role of trial history on performance, and to assess the generality of Masson and Kliegl's (2013) claim that additive effects of word frequency and stimulus quality are a spurious outcome of ignoring trial history. Evaluating the effects of such trial level variables is only possible in LMM and GLMM using unaggregated data because they allow structural dependencies to be accounted for as random factors. However, Balota et al. (2013) reported no evidence of previous trial history significantly modulating the relationship between word frequency and stimulus quality, so these variables were not included in our analyses.

^2^At the time of writing, implementation of LMM and GLMM in popular statistical software assumes that the mean responses across the units of the random factor are normally distributed. Though this may be a reasonable assumption given that sample means can be normally distributed even though the underlying population of responses is non-normal based on the central limit theorem, further advances in computation may allow non-normally distributed random factors to be specified in doubly generalized linear mixed-effect models as described by Lee et al. (2006).

^3^It is important to note that differences in the logs of the means (i.e., passing $\hat{\mu }$ through a log link) is not the same as differences in the means of log-transformed data, but general compression in differences involving larger values on either scale is maintained in either method.

^4^Empirical fit indices such as AIC/BIC values are not comparable across models with different dependent variables (Burnham and Anderson, 2002).

^5^Other more appropriate methods, such as regression splines (Friedman and Roosen, 1995) and generalized additive models (Hastie and Tibshirani, 1990), are available if the goal is estimation of this relationship.
