# Supplementary material for: To transform or not to transform: using generalized linear mixed models to analyse reaction time data
Source: Front Psychol. 2015 Aug 7;6:1171. doi: 10.3389/fpsyg.2015.01171 (PMC4528092; doi:10.3389/fpsyg.2015.01171)
Supplement: Supplementary file 1 [file DataSheet1.DOCX]

**APPENDIX**

All of the statistical models used normalized sum contrasts for the factors of frequency (F) and stimulus quality (Q). Since Low-Frequency words and Clear stimulus conditions received a contrast weight of -0.5, and High-Frequency words and Degraded stimulus conditions a weighting of 0.5, negative estimates for the frequency by stimulus quality interaction (Q:F) denote overadditive effects, while positive estimates denote underadditive effects.

The following R syntax was used to code the specific form of the inverse link function ($-1000/\hat{\mu}$), which was called when generating models assuming an inverse relationship between the predictors and RT.

invfn <- function() {

## link

linkfun <- function(y) -1000/y

## inverse link

linkinv <- function(eta) -1000/eta

## derivative of invlink wrt eta

mu.eta <- function(eta) { 1000/(eta^2) }

valideta <- function(eta) TRUE

link <- "-1000/y"

structure(list(linkfun = linkfun, linkinv = linkinv,

mu.eta = mu.eta, valideta = valideta,

name = link),

class = "link-glm")

}

*Table A.1. R syntax and model output for the Fixed and Random factors of the Yap and Balota (2007) experiment*

|  |  | Gaussian Distribution | | |  | Gamma Distribution | | | |  | Inverse Gaussian Distribution | | | |
| --- | --- | --- | --- | --- | --- | --- | --- | --- | --- | --- | --- | --- | --- | --- |
|  |  | Estimate | Std. Error | t value |  | Estimate | Std. Error | z value | Pr(>\|z\|) |  | Estimate | Std. Error | z value | Pr(>\|z\|) |
| Identity Link | (Intercept) | 664.87 | 12.43 | 53.48 |  | 660.81 | 5.81 | 113.67 | <0.01 |  | 658.64 | 5.52 | 119.32 | <0.01 |
|  | Q | 141.49 | 24.28 | 5.83 |  | 138.71 | 11.08 | 12.52 | <0.01 |  | 136.83 | 10.51 | 13.02 | <0.01 |
|  | F | -55.39 | 6.87 | -8.06 |  | -53.35 | 5.69 | -9.38 | <0.01 |  | -52.4 | 5.48 | -9.57 | <0.01 |
|  | Q:F | -4.45 | 8.6 | -0.52 |  | -4.75 | 8.93 | -0.53 | 0.595 |  | -4.71 | 8.63 | -0.55 | 0.586 |
|  |  |  |  |  |  |  |  |  |  |  |  |  |  |  |
| Inverse Link | (Intercept) | -1.629 | 0.027 | -59.5 |  | -1.56 | 0.01 | -116 | <0.01 |  | -1.56376 | 0.01252 | -124.89 | <0.01 |
|  | Q | 0.319 | 0.053 | 5.98 |  | 0.33 | 0.03 | 12.76 | <0.01 |  | 0.32941 | 0.02376 | 13.86 | <0.01 |
|  | F | -0.135 | 0.015 | -8.91 |  | -0.13 | 0.01 | -9.52 | <0.01 |  | -0.12856 | 0.01337 | -9.62 | <0.01 |
|  | Q:F | 0.042 | 0.018 | 2.39 |  | 0.04 | 0.02 | 1.87 | 0.0608 |  | 0.04024 | 0.02155 | 1.87 | 0.0619 |

|  |  | Gaussian Distribution | |  | Gamma Distribution | |  | Inverse Gaussian Distribution | |
| --- | --- | --- | --- | --- | --- | --- | --- | --- | --- |
|  |  | Variance | Std.Dev. |  | Variance | Std.Dev. |  | Variance | Std.Dev. |
| Identity Link | Item: Intercept | 1675.5 | 40.93 |  | 900.4 | 30.0069 |  | 812.4 | 28.5 |
|  | Item:Q | 973.2 | 31.2 |  | 1130 | 33.6174 |  | 987.6 | 31.43 |
|  | Subject: Intercept | 10363.2 | 101.8 |  | 1972 | 44.4099 |  | 1762 | 41.98 |
|  | Subject:F | 368.8 | 19.2 |  | 508.3 | 22.5453 |  | 477.1 | 21.84 |
|  | Residual | 27369.2 | 165.44 |  | 0.05312 | 0.2305 |  | 0.000078 | 0.008827 |
| Inverse Link | Item: Intercept | 0.008 | 0.0894 |  | 0.0047 | 0.0686 |  | 0.0047 | 0.0686 |
|  | Item:Q | 0.0021 | 0.0454 |  | 0.0066 | 0.0813 |  | 0.0063 | 0.0796 |
|  | Subject: Intercept | 0.0505 | 0.2248 |  | 0.0106 | 0.103 |  | 0.0089 | 0.0945 |
|  | Subject:F | 0.003 | 0.0544 |  | 0.0033 | 0.0577 |  | 0.0035 | 0.0588 |
|  | Residual | 0.0892 | 0.2987 |  | 0.0517 | 0.2274 |  | 0.000076 | 0.0087 |

**Identity Link, Gaussian Distribution**: lmer(RT ~ Q*F +(1|Subject) +(0+F|Subject) +(1|Item) +(0+Q|Item), data=yb07)

**Identity Link, Gamma Distribution**: glmer(RT ~ Q*F +(1|Subject) +(0+F|Subject) +(1|Item) +(0+Q|Item), data=yb07, family=Gamma(link="identity"))

**Identity Link, Inverse Gaussian Distribution**: glmer(RT ~ Q*F +(1|Subject) +(0+F|Subject) +(1|Item) +(0+Q|Item), data=yb07, family=inverse.gaussian(link="identity"))

**Inverse Link, Gaussian Distribution**: lmer(-1000/RT ~ Q*F +(1|Subject) +(0+F|Subject) +(1|Item) +(0+Q|Item), data=yb07)

**Inverse Link, Gamma Distribution**: glmer(RT ~ Q*F +(1|Subject) +(0+F|Subject) +(1|Item) +(0+Q|Item), data=yb07, family=Gamma(link=invfn()))

**Inverse Link, Inverse Gaussian Distribution**: glmer(RT ~ Q*F +(1|Subject) +(0+F|Subject) +(1|Item) +(0+Q|Item), data=yb07, family=inverse.gaussian(link=invfn()))

*Table A.2. R syntax and model output for the Fixed and Random factors of the Yap et al. (2008, Exp 1) experiment*

|  |  | Gaussian Distribution | | |  | Gamma Distribution | | | |  | Inverse Gaussian Distribution | | | |
| --- | --- | --- | --- | --- | --- | --- | --- | --- | --- | --- | --- | --- | --- | --- |
| Link Function | Fixed Factors | Estimate | Std. Error | t value |  | Estimate | Std. Error | z value | Pr(>\|z\|) |  | Estimate | Std. Error | z value | Pr(>\|z\|) |
| Identity Link | (Intercept) | 622.45 | 11.66 | 53.37 |  | 618.84 | 6.53 | 94.76 | <0.01 |  | 616.97 | 6.31 | 97.84 | <0.01 |
|  | Q | 74.62 | 7.56 | 9.87 |  | 72.07 | 7.19 | 10.03 | <0.01 |  | 70.72 | 7.08 | 9.99 | <0.01 |
|  | F | -51.94 | 7.05 | -7.37 |  | -50.02 | 6.95 | -7.2 | <0.01 |  | -49.04 | 6.76 | -7.25 | <0.01 |
|  | Q:F | -9.3 | 8.37 | -1.11 |  | -8.1 | 10.82 | -0.75 | 0.454 |  | -7.63 | 10.62 | -0.72 | 0.472 |
|  |  |  |  |  |  |  |  |  |  |  |  |  |  |  |
| Inverse Link | (Intercept) | -1.704 | 0.028 | -61.13 |  | -1.63 | 0.017 | -96.9 | <0.01 |  | -1.64 | 0.016 | -100.45 | <0.01 |
|  | Q | 0.188 | 0.014 | 13.63 |  | 0.189 | 0.017 | 10.87 | <0.01 |  | 0.188 | 0.017 | 10.89 | <0.01 |
|  | F | -0.135 | 0.015 | -8.75 |  | -0.132 | 0.018 | -7.23 | <0.01 |  | -0.132 | 0.018 | -7.29 | <0.01 |
|  | Q:F | 0.013 | 0.018 | 0.74 |  | 0.008 | 0.029 | 0.26 | 0.795 |  | 0.008 | 0.029 | 0.28 | 0.783 |

|  |  | Gaussian Distribution | |  | Gamma Distribution | |  | Inverse Gaussian Distribution | |
| --- | --- | --- | --- | --- | --- | --- | --- | --- | --- |
| Link Function | Random Factors | Variance | Std.Dev. |  | Variance | Std.Dev. |  | Variance | Std.Dev. |
| Identity Link | Item: Intercept | 1330 | 36.46 |  | 1159 | 34.04 |  | 1106 | 33.25 |
|  | Item: Q | 0 | 0 |  | 1254 | 35.41 |  | 1208 | 34.75 |
|  | Subject: Intercept | 3499 | 59.15 |  | 919.1 | 30.32 |  | 846.5 | 29.09 |
|  | Subject: Q | 1109 | 33.3 |  | 824.7 | 28.72 |  | 795.6 | 28.21 |
|  | Subject: F | 153.4 | 12.39 |  | 258 | 16.06 |  | 226.2 | 15.04 |
|  | Subject: Q:F | 0 | 0.01 |  | 804.1 | 28.36 |  | 759.4 | 27.56 |
|  | Residual | 22510 | 150 |  | 0.0532 | 0.2307 |  | 0.00008 | 0.0092 |
|  |  |  |  |  |  |  |  |  |  |
| Inverse Link | Item: Intercept | 0.0078 | 0.0884 |  | 0.0082 | 0.0903 |  | 0.0081 | 0.0901 |
|  | Item: Q | 0 | 0 |  | 0.0103 | 0.1014 |  | 0.0098 | 0.0992 |
|  | Subject: Intercept | 0.0201 | 0.1418 |  | 0.006 | 0.0777 |  | 0.0056 | 0.0745 |
|  | Subject: Q | 0.0031 | 0.0557 |  | 0.0041 | 0.0637 |  | 0.0041 | 0.0638 |
|  | Subject: F | 0.0000 | 0.0000 |  | 0.0018 | 0.0429 |  | 0.0017 | 0.0414 |
|  | Subject: Q:F | 0 | 0 |  | 0.0062 | 0.0791 |  | 0.0062 | 0.0785 |
|  | Residual | 0.1031 | 0.3211 |  | 0.051489 | 0.22691 |  | 0.00008 | 0.00904 |

**Identity Link, Gaussian Distribution**: lmer(RT ~ Q*F +(1|Subject) +(0+Q|Subject) +(0+F|Subject) +(0+Q:F|Subject) +(1|Item) +(0+Q|Item), data=yb08e1)

**Identity Link, Gamma Distribution**: glmer(RT ~ Q*F +(1|Subject) +(0+Q|Subject) +(0+F|Subject) +(0+Q:F|Subject) +(1|Item) +(0+Q|Item), data=yb08e1, family=Gamma(link="identity"))

**Identity Link, Inverse Gaussian Distribution**: glmer(RT ~ Q*F +(1|Subject) +(0+Q|Subject) +(0+F|Subject) +(0+Q:F|Subject) +(1|Item) +(0+Q|Item), data=yb08e1, family=inverse.gaussian(link="identity"))

**Inverse Link, Gaussian Distribution**: lmer(-1000/RT ~ Q*F +(1|Subject) +(0+Q|Subject) +(0+F|Subject) +(0+Q:F|Subject) +(1|Item) +(0+Q|Item), data=yb08e1)

**Inverse Link, Gamma Distribution**: glmer(RT ~ Q*F +(1|Subject) +(0+Q|Subject) +(0+F|Subject) +(0+Q:F|Subject) +(1|Item) +(0+Q|Item), data=yb08e1, family=Gamma(link=invfn()))

**Inverse Link, Inverse Gaussian Distribution**: glmer(RT ~ Q*F +(1|Subject) +(0+Q|Subject) +(0+F|Subject) +(0+Q:F|Subject) +(1|Item) +(0+Q|Item), data=yb08e1, family=inverse.gaussian(link=invfn()))

*Table A.3. R syntax and model output for the Fixed and Random factors of the Yap et al. (2008, Exp 2) experiment*

|  |  | Gaussian Distribution | | |  | Gamma Distribution | | | |  | Inverse Gaussian Distribution | | | |
| --- | --- | --- | --- | --- | --- | --- | --- | --- | --- | --- | --- | --- | --- | --- |
| Link Function | Fixed Factors | Estimate | Std. Error | t value |  | Estimate | Std. Error | z value | Pr(>\|z\|) |  | Estimate | Std. Error | z value | Pr(>\|z\|) |
| Identity Link | (Intercept) | 702.29 | 14.98 | 46.89 |  | 695.87 | 7.63 | 91.15 | <0.01 |  | 692.42 | 7.2 | 96.17 | <0.01 |
|  | Q | 108.8 | 9.06 | 12.01 |  | 103.59 | 7.68 | 13.49 | <0.01 |  | 100.74 | 7.46 | 13.51 | <0.01 |
|  | F | -44.86 | 8.92 | -5.03 |  | -47.77 | 7.47 | -6.39 | <0.01 |  | -46.53 | 7.14 | -6.51 | <0.01 |
|  | Q:F | -1.17 | 8.93 | -0.13 |  | -4.12 | 9.79 | -0.42 | 0.674 |  | -4.65 | 9.5 | -0.49 | 0.625 |
|  |  |  |  |  |  |  |  |  |  |  |  |  |  |  |
| Inverse Link | (Intercept) | -1.555 | 0.026 | -58.77 |  | -1.47 | 0.015 | -101.43 | <0.01 |  | -1.475 | 0.014 | -107.24 | <0.01 |
|  | Q | 0.215 | 0.011 | 19.04 |  | 0.215 | 0.014 | 15.78 | <0.01 |  | 0.215 | 0.014 | 15.8 | <0.01 |
|  | F | -0.079 | 0.013 | -6.03 |  | -0.1 | 0.014 | -7.31 | <0.01 |  | -0.1 | 0.014 | -7.23 | <0.01 |
|  | Q:F | 0.025 | 0.076 | 0.33 |  | 0.031 | 0.021 | 1.47 | 0.141 |  | 0.027 | 0.021 | 1.31 | 0.191 |

|  |  | Gaussian Distribution | |  | Gamma Distribution | |  | Inverse Gaussian Distribution | |
| --- | --- | --- | --- | --- | --- | --- | --- | --- | --- |
| Link Function | Random Factors | Variance | Std.Dev. |  | Variance | Std.Dev. |  | Variance | Std.Dev. |
| Identity Link | Item: Intercept | 3058 | 55.3 |  | 1711 | 41.37 |  | 1544 | 39.29 |
|  | Item: Q | 1205 | 34.71 |  | 1882 | 43.38 |  | 1772 | 42.09 |
|  | Subject: Intercept | 11490 | 107.2 |  | 2592 | 50.91 |  | 2277 | 47.72 |
|  | Subject: Q | 3393 | 58.25 |  | 2026 | 45.01 |  | 1879 | 43.34 |
|  | Subject: F | 1814 | 42.59 |  | 1206 | 34.73 |  | 1041 | 32.27 |
|  | Subject: Q:F | 0 | 0 |  | 890.2 | 29.84 |  | 792.1 | 28.14 |
|  | Residual | 39140 | 197.8 |  | 0.07 | 0.26 |  | 0.0001 | 0.0099 |
|  |  |  |  |  |  |  |  |  |  |
| Inverse Link | Item: Intercept | 0.0113 | 0.1064 |  | 0.0072 | 0.0849 |  | 0.0072 | 0.0849 |
|  | Item: Q | 0.0045 | 0.0673 |  | 0.0098 | 0.0989 |  | 0.0095 | 0.0977 |
|  | Subject: Intercept | 0.0355 | 0.1883 |  | 0.0089 | 0.0945 |  | 0.0078 | 0.0884 |
|  | Subject: Q | 0.0036 | 0.0599 |  | 0.0045 | 0.0672 |  | 0.0047 | 0.0683 |
|  | Subject: F | 0.0018 | 0.0428 |  | 0.003 | 0.0548 |  | 0.0031 | 0.0557 |
|  | Subject: Q:F | 0.3125 | 0.559 |  | 0.0048 | 0.0692 |  | 0.005 | 0.0709 |
|  | Residual | 0.1035 | 0.3216 |  | 0.0673 | 0.2594 |  | 0.0001 | 0.0097 |

**Identity Link, Gaussian Distribution**: lmer(RT ~ Q*F +(1|Subject) +(0+Q|Subject) +(0+F|Subject) +(0+Q:F|Subject) +(1|Item) +(0+Q|Item), data=yb08e2)

**Identity Link, Gamma Distribution**: glmer(RT ~ Q*F +(1|Subject) +(0+Q|Subject) +(0+F|Subject) +(0+Q:F|Subject) +(1|Item) +(0+Q|Item), data=yb08e2, family=Gamma(link="identity"))

**Identity Link, Inverse Gaussian Distribution**: glmer(RT ~ Q*F +(1|Subject) +(0+Q|Subject) +(0+F|Subject) +(0+Q:F|Subject) +(1|Item) +(0+Q|Item), data=yb08e2, family=inverse.gaussian(link="identity"))

**Inverse Link, Gaussian Distribution**: lmer(-1000/RT ~ Q*F +(1|Subject) +(0+Q|Subject) +(0+F|Subject) +(0+Q:F|Subject) +(1|Item) +(0+Q|Item), data=yb08e2)

**Inverse Link, Gamma Distribution**: glmer(RT ~ Q*F +(1|Subject) +(0+Q|Subject) +(0+F|Subject) +(0+Q:F|Subject) +(1|Item) +(0+Q|Item), data=yb08e2, family=Gamma(link=invfn()))

**Inverse Link, Inverse Gaussian Distribution**: glmer(RT ~ Q*F +(1|Subject) +(0+Q|Subject) +(0+F|Subject) +(0+Q:F|Subject) +(1|Item) +(0+Q|Item), data=yb08e2, family=inverse.gaussian(link=invfn()))

*Table A.4. Model estimates for each Frequency by Stimulus Quality condition back-transformed onto the original RT scale assuming an identity or inverse link function, or a Gaussian, Gamma or Inverse Gaussian distribution of RT.*

|  |  |  | Identity Link | | |  | Inverse Link | | |
| --- | --- | --- | --- | --- | --- | --- | --- | --- | --- |
| Experiment | Stimulus Quality | Frequency | Gaussian | Gamma | Inverse Gaussian | | Gaussian (invRT) | Gamma | Inverse Gaussian |
| Yap & Balota (2007) | Clear | High | 568 | 566 | 565 |  | 536 | 556 | 555 |
|  |  | Low | 621 | 617 | 615 |  | 585 | 606 | 605 |
|  | Degraded | High | 707 | 702 | 700 |  | 655 | 690 | 688 |
|  |  | Low | 764 | 758 | 754 |  | 708 | 745 | 744 |
|  |  |  |  |  |  |  |  |  |  |
| Yap et al.  (2008, Exp 1) | Clear | High | 562 | 560 | 559 |  | 535 | 558 | 556 |
|  |  | Low | 609 | 606 | 604 |  | 579 | 603 | 602 |
|  | Degraded | High | 631 | 628 | 626 |  | 597 | 625 | 623 |
|  |  | Low | 688 | 682 | 679 |  | 647 | 679 | 677 |
|  |  |  |  |  |  |  |  |  |  |
| Yap et al.  (2008, Exp 2) | Clear | High | 626 | 621 | 620 |  | 585 | 611 | 610 |
|  |  | Low | 670 | 667 | 664 |  | 618 | 658 | 655 |
|  | Degraded | High | 734 | 723 | 718 |  | 675 | 712 | 709 |
|  |  | Low | 779 | 773 | 767 |  | 707 | 757 | 755 |
